# Supplementary figures and images for: Prolonged overexpression of PLK4 leads to formation of centriole rosette clusters that are connected via canonical centrosome linker proteins
Source: Sci Rep. 2024 Feb 22;14:4370. doi: 10.1038/s41598-024-53985-2 (PMC10883960; doi:10.1038/s41598-024-53985-2)

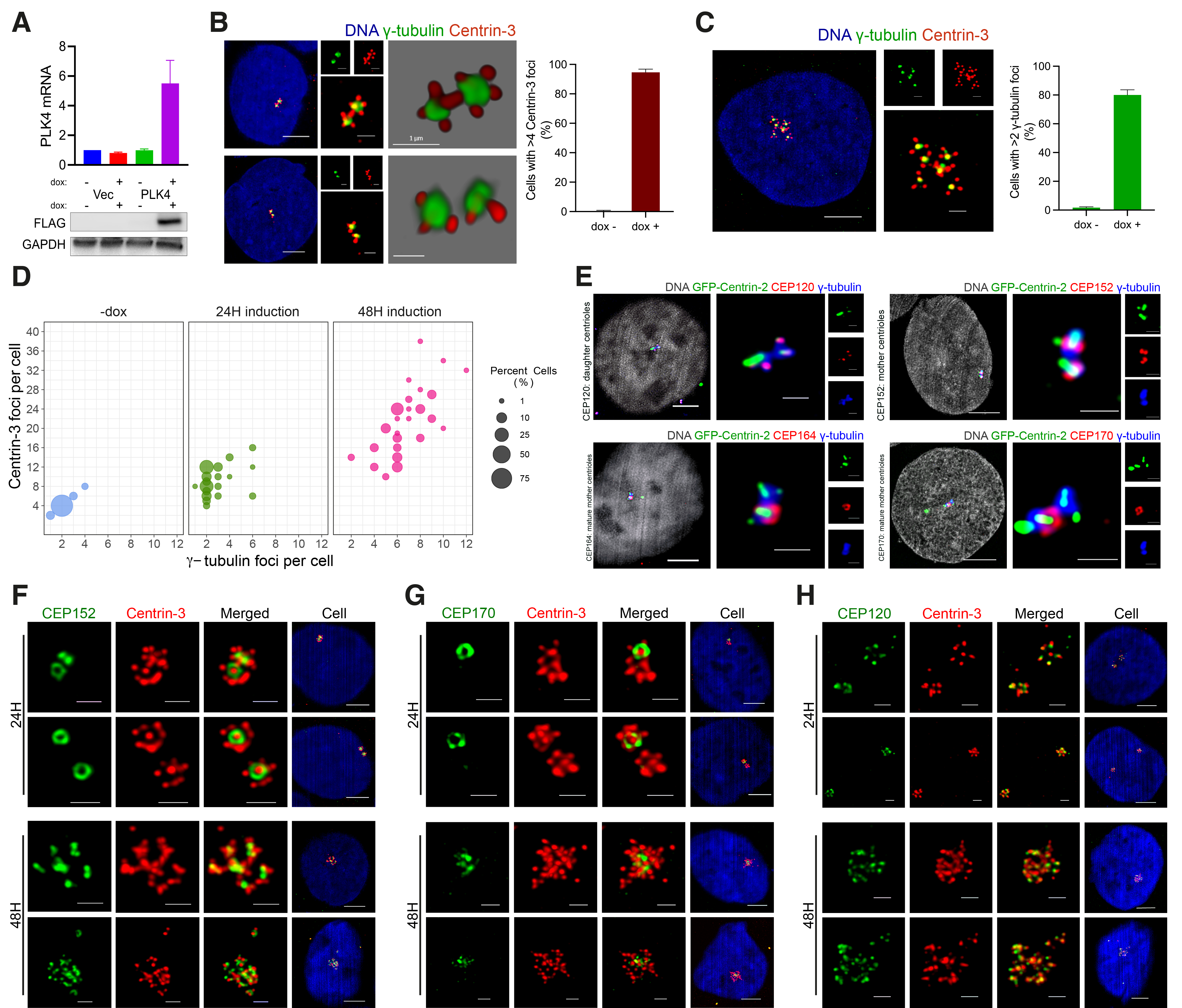

Supplement: Supplementary file 1 — Supplementary Figure S1. [file 41598_2024_53985_MOESM1_ESM.tif]

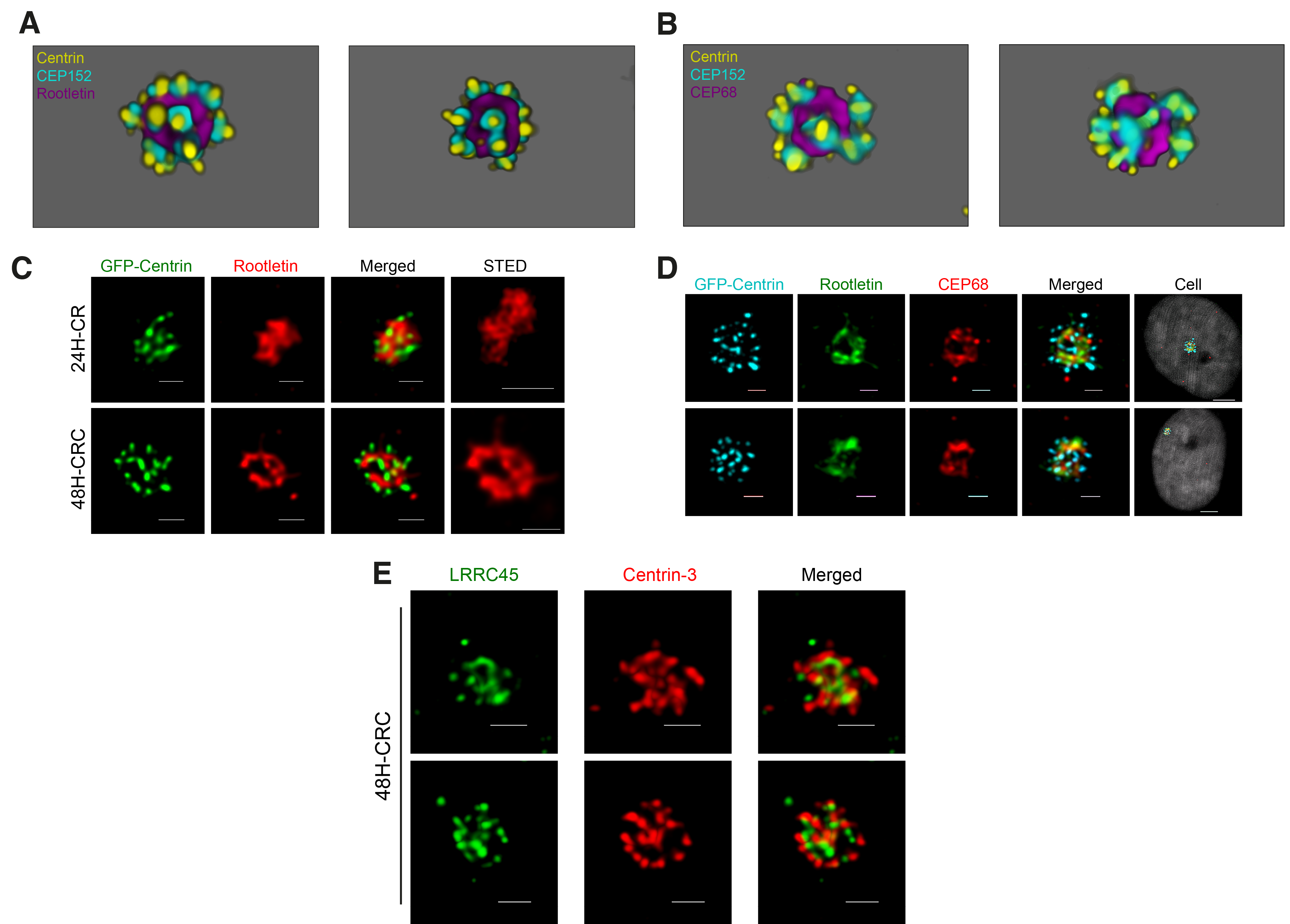

Supplement: Supplementary file 2 — Supplementary Figure S2. [file 41598_2024_53985_MOESM2_ESM.tif]

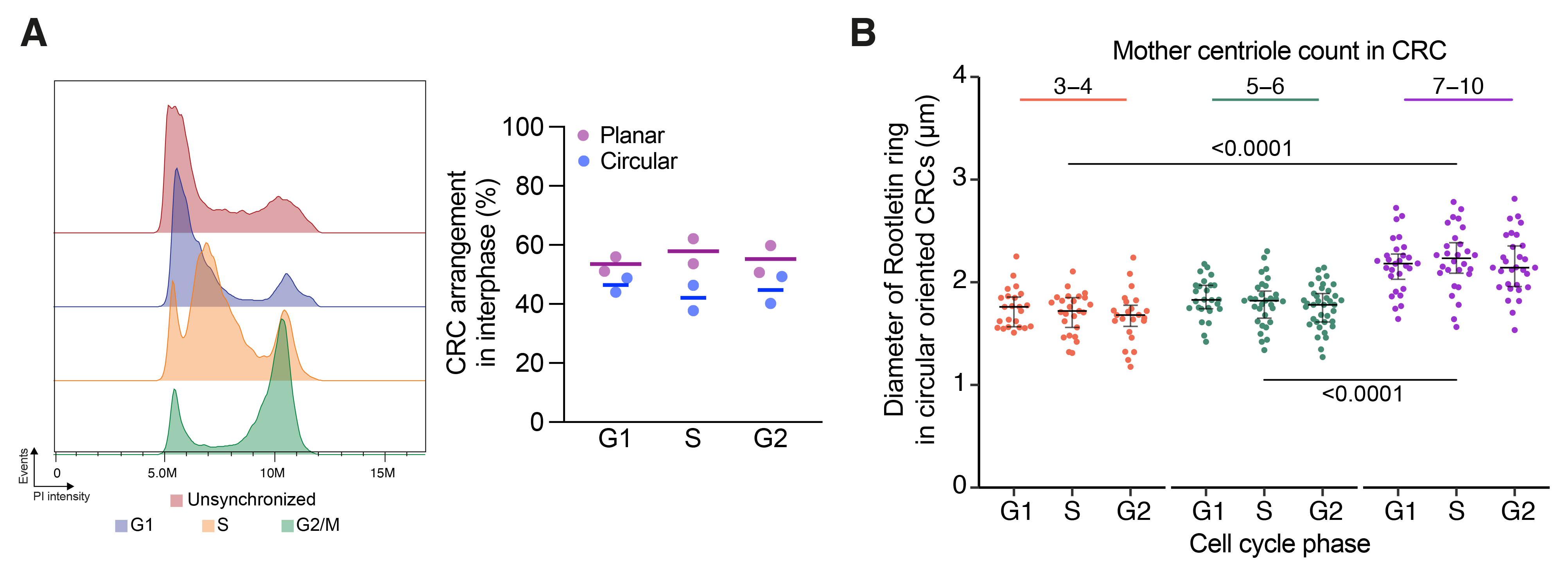

Supplement: Supplementary file 3 — Supplementary Figure S3. [file 41598_2024_53985_MOESM3_ESM.tif]

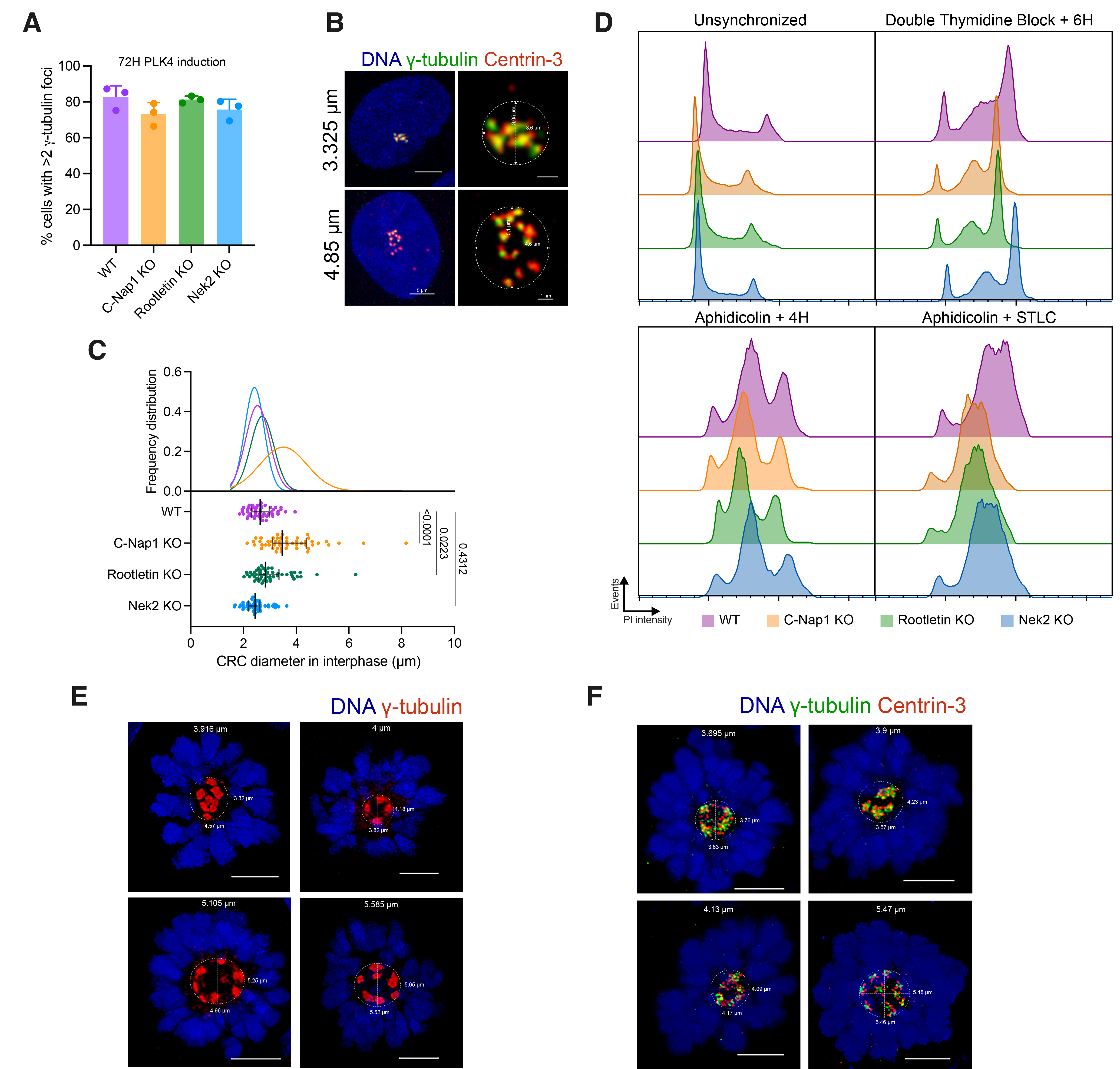

Supplement: Supplementary file 4 — Supplementary Figure S4. [file 41598_2024_53985_MOESM4_ESM.tif]

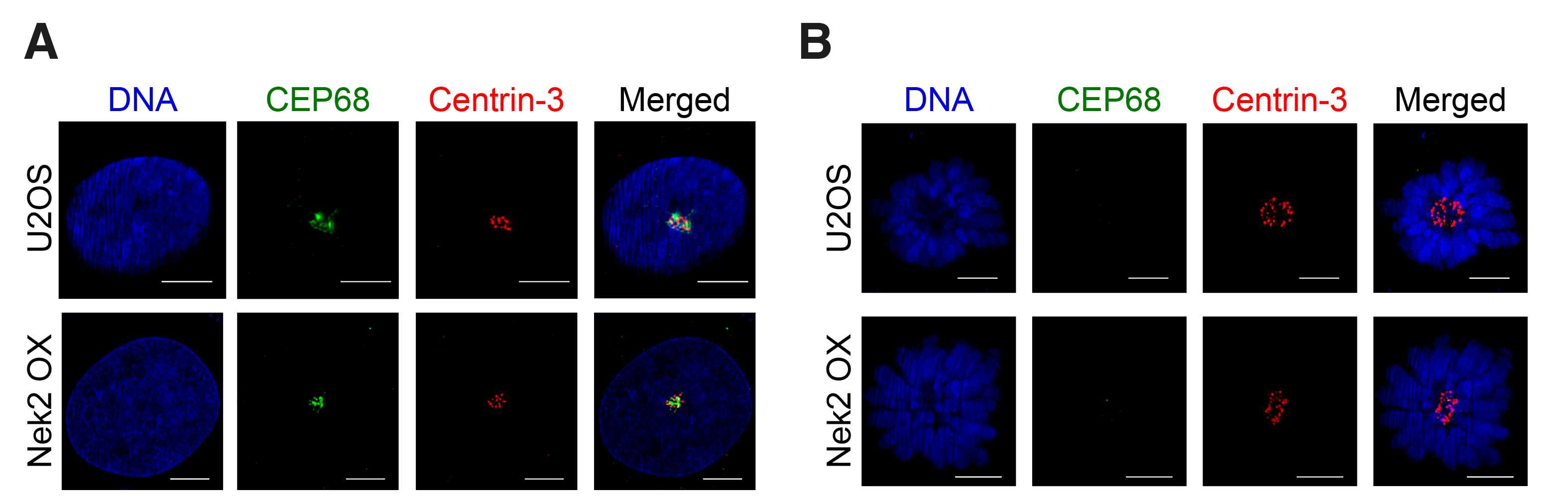

Supplement: Supplementary file 5 — Supplementary Figure S5. [file 41598_2024_53985_MOESM5_ESM.tif]

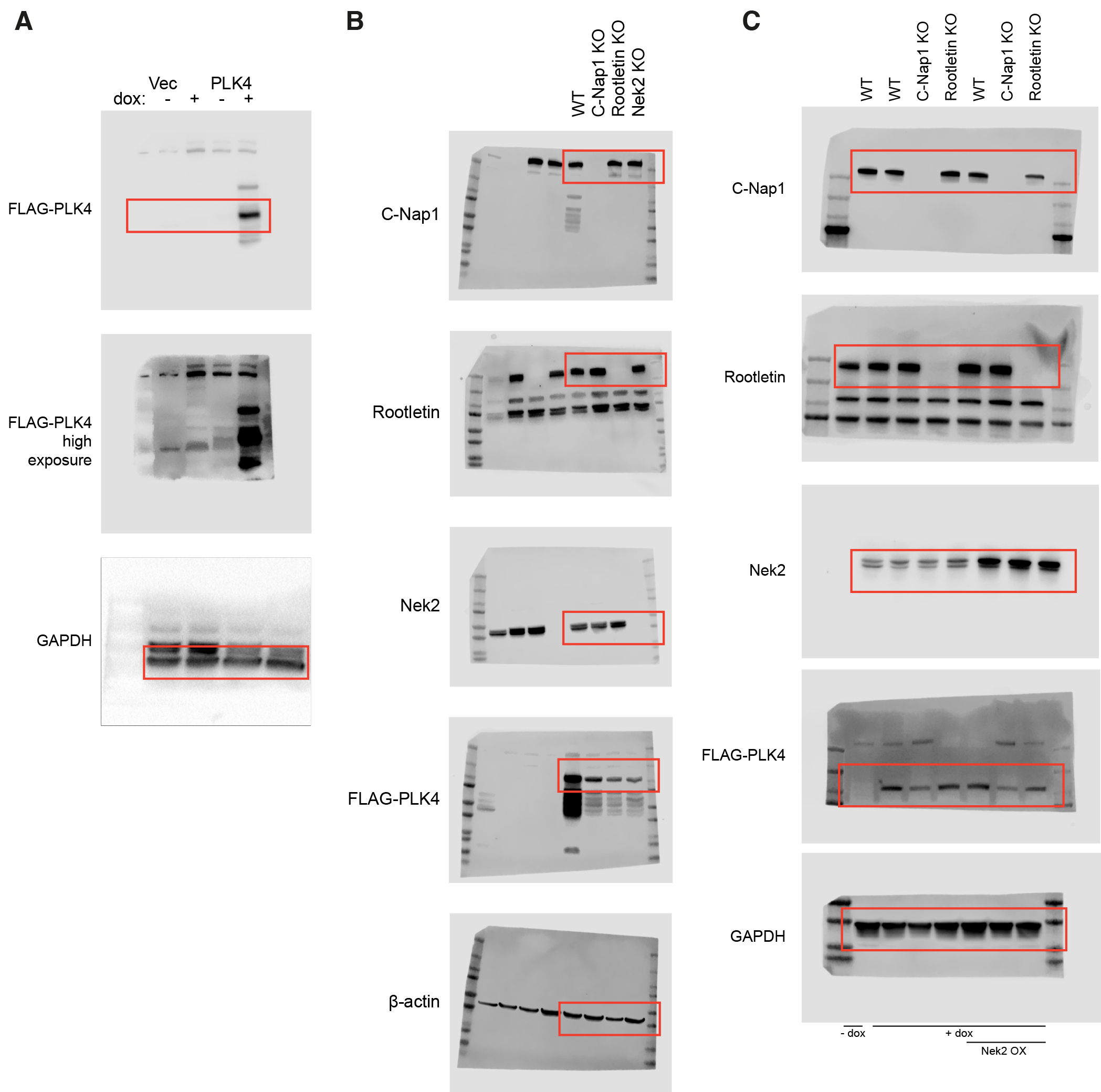

Supplement: Supplementary file 6 — Supplementary Figure S6. [file 41598_2024_53985_MOESM6_ESM.tif]
