# Supplementary material for: Prolonged overexpression of PLK4 leads to formation of centriole rosette clusters that are connected via canonical centrosome linker proteins
Source: Sci Rep. 2024 Feb 22;14:4370. doi: 10.1038/s41598-024-53985-2 (PMC10883960; doi:10.1038/s41598-024-53985-2)
Supplement: Supplementary file 7 — Supplementary Legends. [file 41598_2024_53985_MOESM7_ESM.pdf]

Supplementary Figures

Supplemental Figure 1 **Supplemental to Fig. 1** A) Doxycycline induced PLK4 over-expression. B) 24-hours PLK4 induction generates CRs. Left panel: Two cells with CRs. Cells were stained with DAPI (DNA, blue), γ-tubulin (centrosome, green) and Centrin-3 (centriole, red). Right panel: Percentage of cells with >4 Centrin-3 foci in -dox and +dox conditions. C) 48-hours PLK4 induction forms CRCs. Left panel: Two cells with CRCs. Cells were stained with DAPI (DNA, blue), γ-tubulin (centrosome, green) and Centrin-3 (centriole, red). Right panel: Percentage of cells with >2 γ-tubulin foci in -dox and +dox conditions. D) Quantification of centrosomes and total centriole numbers in 24-hours and 48-hours PLK4 induced cells. n:100, N:2. Raw measurement data is available in Supplemental Table 1. E) Co-staining of CEP120 / CEP152 / CEP164 and CEP170 with γ-tubulin in GFP-Centrin-2 expressing U2OS cells. F-H) Representative images of localization of CEP152 (F), CEP170 (G) and CEP120 (H) in 24-hours (upper panels) and 48-hours (bottom panels) dox induced cells.

Supplemental Figure 2 **Supplemental to Fig. 2** A-B) 3D visualization of Z-stacks from Figure 2C. (A) and Figure 2D (B). C) Representative confocal and STED images showing the connection of CRs and CRCs with Rootletin. D) Representative confocal images of co-staining of Rootletin and CEP68 in cells with CRCs. E) LRRC45 localization in CRCs.

Supplemental Figure 3 **Supplemental to Fig. 3** A) Left panel: Cell cycle synchronization with double thymidine block, Right panel: Percentage of CRC arrangement type in cells with CRCs. Dots represent biological repeats, and lines display the mean of repeats.G1 phase: 0h after DTB, S phase: 4h after DTB release, G2/M phase: 8h after DTB release. B) Diameter of Rootletin ring in circular linked CRCs is not changed with cell cycle progression (n; G1: 77, S: 84, G2: 90 circular linked CRCs from 2 independent experiments.). Lines represent median and interquartile range. Raw measurement data of S3A and S3B are available in Supplemental Table 2.

Supplemental Figure 4 **Supplemental to Fig. 4** A) Centrosome amplification in 72 hours PLK4 induced U2OS-WT and U2OS-KO cells. B) Representative measurements of two cells with CRCs (blue: DAPI, green: γ-tubulin, red: Centrin-3. Note that γ-tubulin negative disengaged centrioles in bottom image are not included in diameter measurements.) C) CRC diameter in 48 hours PLK4 induced unsynchronized cells. Median and interquartile range is shown on plot. n: 50 for each group, pooled from 2 independent experiment. Raw measurement data is available in Supplemental Table 3. D) Cell cycle profiles of unsynchronized and synchronized cells. E) Representative images of prometaphase distances in cells with CRCs. Cells were stained with DAPI (DNA, blue) and γ-tubulin (centrosome, red). F) Representative images of prometaphase distances in cells with CRCs. Cells were stained with DAPI (DNA, blue), γ-tubulin (centrosome, green) and Centrin-3 (centriole, red).

Supplemental Figure 5 **Uncropped Western blots** Uncropped western blot images of Fig. S1A (A), Fig. 4A (B), Fig. 5A (C). Red rectangular indicates the parts of the blots that are used in figures.

Supplementary Tables

Supplemental Table 1 Scoring data of Centrin-3 foci vs. CEP152, CEP170 and γ-tubulin foci of -dox, 24H PLK4 induced and 48H PLK4 induced cells in Fig. S1D, Fig. 1E and Fig. 1F.

Supplemental Table 2 Scoring data of planar vs. circular oriented CRCs presented in Figs. 3B, 3C, S3A, 3D and S3B.

Supplemental Table 3 Scoring data of CR and CRCs distances in Figs. S4C, 4C, 4D, 4E, 5B and 5C.

Supplemental Table 4 Scoring data of Centrin-3 foci vs. CEP152 foci in Fig. 6C.
